# Maternal Functioning and Child's Externalizing Problems: Temperament and Sex-Based Driven Effects

**DOI:** 10.3389/fpsyg.2022.874733

**Published:** 2022-05-17

**Authors:** Gabrielle Garon-Carrier, Katherine Pascuzzo, William Gaudreau, Jean-Pascal Lemelin, Michèle Déry

**Affiliations:** ^1^Département de Psychoéducation, Université de Sherbrooke, Sherbrooke, QC, Canada; ^2^Groupe de Recherche et d'Intervention sur les Adaptations Sociales de l'Enfance de l'Université de Sherbrooke (GRISE), Université de Sherbrooke, Sherbrooke, QC, Canada

**Keywords:** psychological distress, neglect, hostility, warmth, temperament, sex differences, externalizing problems

## Abstract

This study examines how maternal adverse parenting (hostility, neglect, low warmth) and psychological distress explain the associations between child temperament factors and externalizing problems. It also examines if these associations differ according to the child's biological sex. The sample consists of 339 school-age children receiving in-school services for conduct problems. Data were collected through questionnaires completed by mothers at 3 time points, at one-year intervals. Results from path analyses revealed that maternal psychological distress partly explained the associations between each child temperamental factors (negative affectivity, surgency/extraversion, effortful control) and levels of externalizing problems. Specifically, the indirect effect of psychological distress between child negative affectivity and externalizing problems was only significant for boys, not girls. Maternal hostility, on the other hand, mediated the association between child surgency/extraversion and externalizing problems in both boys and girls. Interestingly, neglectful parenting and maternal warmth did not explain the association between child temperamental factors and externalizing problems. The findings suggest small but significant temperament child-driven effects on maternal psychological distress and hostility, in turn, translating into higher levels of externalizing problems. These findings support the relevance of temperament-based interventions for children with conduct problems and of increased mental health support for their mothers. By aiding mothers in developing a larger repertoire of parenting strategies, mothers may be better equipped to respond appropriately to their child's various temperamental characteristics, hence, reducing their psychological distress and hostile behaviors and limiting the development of child externalizing problems.

## Introduction

Children exhibiting externalizing problems, characterized by non-compliance to rules and aggression, represent the majority of referrals to elementary school-based mental health services (Burnett-Zeigler and Lyons, 2012; Briesch et al., 2013). In the US, the prevalence of children with externalizing problems ranges between 1% and 10%, with longitudinal and epidemiological studies consistently showing a greater proportion of boys than girls displaying such behaviors (Berkout et al., 2011; American Psychiatric Association., 2013). These children often experience chronic difficulties in both academic and social domains (Deighton et al., 2018), which evolve into more severe problems in the absence of intervention, including juvenile delinquency and adult crime (Wertz et al., 2018).

In an effort to prevent the development of these behaviors, many scholars have investigated the child-context interplay leading to externalizing problems. An approach that has received great attention over the last decade and has guided research on this dynamic interplay is the transactional perspective (Sameroff, 2009). Central to this perspective is the bidirectional and interdependent association between children and their social context. To guide our understanding of the link between child temperamental factors and the parenting context, this perspective would suggest that a child with a higher level of irritability could elicit lower levels of maternal warmth, which would subsequently lead to increased child externalizing problems.

Another theoretical perspective focusing on the child-context interplay to understand the development of externalizing problems is the differential susceptibility model to environmental influences (Belsky, 2013; Slagt et al., 2016). According to this model, specific dispositional traits could place children at greater risk for negative outcomes when confronted with poorer parenting. Conversely, these same traits could enhance adaptation under positive parenting circumstances. This model has received empirical support, showing differential susceptibility to externalizing behavior at age 12 among children presenting negative affectivity, depending on parenting quality (Stoltz et al., 2017).

Stemming from the transactional (Sameroff, 2009) and the differential susceptibility (Belsky, 2013) perspectives, the present study focuses on child-context interplay leading to externalizing problems. More specifically, we sought to examine how child temperamental factors (individual dispositional traits) are associated with adverse maternal parenting (hostility, neglect, low warmth) and psychological distress (family-wide context) and explain child levels of externalizing problems.

### Child Temperament as Dispositional Traits to Externalizing Problems

Child temperament refers to individual differences in reactivity as expressed at the emotional, attentional, and motor levels (negative affectivity, surgency/extraversion), and in the ability to regulate reactivity (effortful control) (Putnam and Stifter, 2008; Rothbart, 2012). These individual differences emerge early in children's lives, have a biological base, and are relatively stable across time and contexts (Rothbart, 2012). Negative affectivity is the child's tendency to react to new, unpleasant or potentially threatening situations with various negative emotions (e.g., fear, anger, sadness), and to be difficult to soothe. Surgency/extraversion reflects the child's levels of sociability, impulsivity and activity, as well as their propensity to seek sensations. Lastly, effortful control refers to the child's ability to focus attention and inhibit inappropriate behaviors.

Studies have consistently and reliably shown that children presenting temperamental vulnerability for psychopathology, characterized by high negative affectivity and surgency/extraversion and low effortful control, are at greater risk of externalizing problems (Nielsen et al., 2019). For instance, higher surgency and negative affectivity, such as anger proneness, have been associated with an increased risk for externalizing behaviors (Scheper et al., 2017; Sirois et al., 2019). Effortful control also plays a role in shaping both externalizing and internalizing problems (Scheper et al., 2017), with stronger associations reported for externalizing difficulties (Liu et al., 2020). Moreover, sex differences in temperamental characteristics revealed that girls are less likely to present temperamental susceptibility to externalizing problems. Based on the findings of a meta-analytic review, girls exhibit higher levels of regulatory ability than boys (Else-Quest et al., 2006), which could partly explain the lower prevalence of externalizing problems among girls than boys.

### Parenting as Contextual Factors to Externalizing Problems

Parenting behaviors such as maternal hostility, physical and emotional neglect, low levels of warmth/sensitivity, and psychological distress (e.g., anxiety, depressive symptoms) have been consistently associated with child externalizing problems, and to a lesser extent, with child internalizing problems (Pinquart, 2017; Hecker et al., 2019; Bellina et al., 2020; Khoury et al., 2021; Yan et al., 2021). Indeed, hostile parenting and psychological distress have been more systematically linked with externalizing problems compared to internalizing problems (Stone et al., 2016; Khoury et al., 2021; Yan et al., 2021). Research underscoring the interlock between maternal psychological distress and child externalizing problems (Yan et al., 2021) suggests that maternal psychological distress is associated with more erratic and unpredictable parenting behaviors (Dubois-Comtois et al., 2013), which could amplify child externalizing problems. As for hostile parenting, it has been more strongly associated with externalizing problems compared to neglect or low warmth/sensitivity (Pinquart, 2017; Khoury et al., 2021). Some studies have also revealed sex-based differences, showing hostile parenting and psychological distress to predict greater externalizing problems for girls, but not for boys (e.g., Burnette et al., 2012). Other studies, however, provide no such evidence (e.g., Yan et al., 2021).

### Child Temperament as a Predictor of Parenting: A Child-Driven Perspective

Child temperamental factors are known predictors of parenting behaviors (Liu et al., 2020). Parents' capacity to manage the child's temper may be undermined in two ways when confronted with a child presenting difficulties in regulating behaviors and emotions. First, the parent may exhibit adverse parenting including hostility and coercion (Silinskas et al., 2015), and lower warmth/sensitivity toward the child (Harvey and Metcalfe, 2012). Second, the parent may exhibit greater psychological distress resulting in increased stress and depressive symptoms (Choe et al., 2014). These findings support the view that child temperament may act as a dual risk factor for adverse parenting and psychological distress. It also suggests that child temperament, as a child-driven effect, could predict externalizing problems through its effects on parenting. Indeed temperament has been shown to have direct effects on the development of externalizing problems (Scheper et al., 2017; Nielsen et al., 2019; Sirois et al., 2019), but also indirect effects through selection or structuring of the environment, eliciting different patterns of parenting (Liu et al., 2020). By ignoring this potential child-driven effect, the impact of adverse parenting and psychological distress on child externalizing problems might have been overestimated in previous studies, at least to some extent.

This study investigates the transactional associations by which maternal hostility, neglect, warmth, and psychological distress explain the association between child temperament factors and levels of externalizing problems. Given that parenting variables (hostility, neglect, and warmth) include a relational dimension with the child, whereas psychological distress is person specific, maternal adverse parenting variables and maternal distress were treated separately in this study. This choice was further guided by recent findings underscoring that parenting and psychological distress are distinctively linked to child adaptation (Khoury et al., 2021).

Based on prior research, we expect that high reactivity and low regulatory abilities will be associated with more adverse parenting and psychological distress. We also expect maternal adverse parenting (especially hostility) and psychological distress to mediate the associations between child temperamental factors and externalizing problems. This study also tests the differential susceptibility of boys and girls to elicit adverse maternal parenting and psychological distress, by examining if the sex of the child moderates the associations between temperamental factors and adverse parenting and distress in the prediction of externalizing problems. Considering that girls are less likely than boys to present temperamental risk for externalizing problems (Else-Quest et al., 2006), variations in the propensity to elicit specific parenting behaviors are expected. At last, the current study seeks to expand the current state of knowledge by examining these mediational effects among a clinically relevant population of boys and girls with conduct problems. While most studies have drawn conclusions from children in the general population (e.g., Nielsen et al., 2019), this study rests on an early-onset clinical sample of school-aged children and overcomes limitations of sex-based differences in the prevalence of externalizing problems among children.

## Methods

### Participants

Participants were part of an ongoing longitudinal study aiming to understand the development, persistence, and consequences of conduct problems throughout childhood and adolescence as a function of child sex/gender (*N* = 744). Children under the age of 10 years (with and without conduct problems) were recruited in three cohorts with the help of eight French-speaking school boards from four administrative regions in the province of Quebec (Estrie, Montérégie, Montréal, and Capitale-Nationale) in Canada between 2008 and 2010.

The recruitment process targeted children receiving psychosocial services for conduct problems in public schools. This is considered an ecologically valid method of recruitment since 95% of children in Quebec attend public elementary schools (Government of Quebec, 2013), and only children with a formal assessment of conduct problems by professionals (e.g., school psychologists) can receive psychosocial services in school. Additionally, children had to reach the borderline clinical cut-off (*T*-score ≥ 65) on the DSM-oriented scales for conduct problems and oppositional defiant problems (Achenbach and Rescorla, 2001) based on parent and teacher reports. Children with an intellectual or sensory disability or a pervasive developmental disorder, as indicated by an administrative code informing on whether the child received a formal diagnosis, were excluded from the study. To ensure an equal proportion of participating boys and girls with conduct problems, all girls receiving services at school for conduct problems and approximately one out of four boys receiving these services (randomly selected) were recruited to participate in the study (*n* = 339; 41.0% of girls). Further details on the recruitment and procedure of this longitudinal study can be found in Boutin et al. (2020).

The current study draws on data collected from children with conduct problems assessed yearly over a 3-year period reflecting three waves of data collection: T1 (*n* = 339), T2 (*n* = 311) and T3 (*n* = 308). The proportion of missing data ranged from 0.1% to 9.1%, with a low yearly attrition rate of 3.0% across the three-time points. Missing data were examined with the Missing Value Analysis module in SPSS. According to Little's missing completely at random (MCAR) test, data were missing completely at random (χ^2^ = 93.79, df = 82, *p* = 0.176), suggesting that children did not differ according to whether they had missing data or not.

### Procedure

Data were collected through questionnaires reported by mothers prior to the COVID-19 pandemic. All the questionnaires were administered in French. Child temperament was measured at T1 (*M* = 8.50, *SD* = 0.93), maternal adverse parenting and psychological distress were assessed at T2 (*M* = 9.41, *SD* = 0.96) and child level of externalizing problems was collected at T3 (*M* = 10.38, *SD* = 0.94). All covariates were also measured through questionnaire at T1.

### Measures

#### Child Externalizing Problems

Externalizing behaviors were assessed using the rule-breaking behaviors scale (e.g., “steals outside the home”) and the aggressive behaviors scale (e.g., “cruelty, bullying, or meanness”) of the Child Behavior Checklist (CBCL/6-18; Achenbach and Rescorla, 2001). We used a French-Canadian translation of the CBCL/6-18, along with the original norms and standards (Achenbach et al., 2003). Mothers rated 35 items on a 3-point Likert scale from 0 (not true) to 2 (very true or often true) and the items were summed. The reliability estimate indicates a satisfactory internal consistency of 0.89. *T*-scores were used in the analyses, with higher scores indicating higher levels of *externalizing behaviors*.

#### Child Temperament

Temperamental factors were evaluated using the French version of Children's Behavior Questionnaire—Short Form (CBQ-SF; Lemelin et al., 2020). Items were rated by the mother on a 7-point Likert scale ranging from 0 (extremely false) to 6 (extremely true): *negative affectivity* (31 items, “Has temper tantrums when she/he doesn't get what he/she wants”; α = 0.84); *surgency/extraversion* (25 items, “Usually rushes into an activity without thinking about it”; α = 0.85); and *effortful control* (26 items, “Can lower his/her voice when asked to do so”; α = 0.76). A total mean score for each temperamental factor was computed with higher scores indicating higher levels of the given trait.

#### Maternal Parenting

The Parental Acceptance-Rejection Questionnaire (PARQ; Rohner, 2005) is a self-report questionnaire designed to assess the mother's perceptions of acceptance and rejection of her child. Three scales of the French version of the PARQ were used to measure *hostility* (15 items, “I hit my child even when he/she may not deserve it”; α = 0.82), *neglect* (15 items, “I pay no attention to my child”; α = 0.71), and low *warmth* (20 items, “I say nice things about my child”; α = 0.87). Items were rated on a 4-point Likert scale from 1 (almost always true) to 4 (almost never true). A total sum score for each scale was computed with higher scores indicating higher levels of the given dimension.

#### Maternal Psychological Distress

Maternal *psychological distress* was measured using a French version of the Psychiatric Symptom Index (Boyer et al., 1993). This self-reported questionnaire, consisting of 14 items, estimates the frequency with which the mother has experienced symptoms of psychological distress (depression, anxiety, irritability, and cognitive problems) over the last 7 days (e.g., During the last week, how often did you: “feel nervous or shaky inside,” “cry easily or feel like crying”). Items were rated on a 4-point Likert scale ranging from 1 (never) to 4 (very often). The internal consistency of this scale was excellent, α = 0.90. A total sum score was computed with a higher score indicating greater levels of psychological distress.

#### Confounding Variables

Among participating children, 74.0% were taking medication for their behavioral difficulties. Approximately 21.0% and 26.0% of children were living with one family member with alcohol or drug problems, respectively. About one third of children (34.5%) came from low-income families (< $30,000/year), 42.5% were from middle-income families ($30,000 to $69 999$/year) and 23.0% of children were from high-income families ($70,000/year or more). These variables were controlled in our analyses, in addition to the age of the child at T1 and the child's initial level of externalizing problems at T1.

### Analytic Strategy

First, we tested the extent to which the associations between temperamental factors (negative affectivity, surgency/extraversion, effortful control) and externalizing problems were mediated by maternal hostility, neglect, warmth, and psychological distress. A total of 12 mediation models were conducted. Child age, initial level of externalizing problems, and medication usage, as well as family income and history of drug and alcohol problems were controlled for in the analyses. The indirect effects were tested with bias-corrected bootstrapping (*n* = 1,000), which does not require the assumption of normal distribution (Preacher et al., 2007). The 95% confidence intervals (CI) of the indirect effect parameter indicates statistical significance. Second, we examined if the sex of the child moderated the associations between child temperament and maternal parenting and psychological distress. When significant, the subgroup method and bootstrapping were applied, which test the mediation effect separately at each level of the moderator. Each model was tested through path analysis using Mplus 7.4. The full information maximum likelihood was used to provide parameter estimates even in the presence of missing data. The model fit was determined using the comparative fit index (CFI, good at 0.95 or above), the Tucker-Lewis Fit index (TLI, acceptable at >0.95), and the root mean square error of approximation (RMSEA, acceptable at 0.06 or below) (Hu and Bentler, 1999).

## Results

Descriptive statistics presented separately for boys and girls are provided in Table 1. On average, girls had higher levels of negative affectivity and effortful control than boys. No significant differences between boys and girls were found on externalizing behaviors or any of the maternal parenting and psychological distress measures. Correlations presented in Table 2 show significant associations between measures. Interestingly, temperamental factors of negative affectivity, surgency/extraversion, and effortful control were not significantly correlated with one another.

**Table 1:** Descriptive statistics, for boys and girls, on main study variables.

	**Mean (SD)**		***t-*** **test**	
	**Boys *n* = 200**	**Girls *n* = 139**	***t***	***p***
**Child temperament T1**				
Surgency/extraversion	4.98 (0.83)	4.92 (0.87)	0.62	0.535
Effortful control	**4.72 (0.67)**	**4.92 (0.57)**	**-2.94**	**0.003**
Negative affectivity	**4.33 (0.76)**	**4.55 (0.81)**	**-2.46**	**0.015**
**Maternal parenting T2**				
Hostility	24.60 (5.59)	25.45 (5.78)	−1.30	0.196
Neglect	19.82 (4.18)	20.23 (3.84)	−0.87	0.385
Warmth	74.69 (5.99)	74.50 (4.39)	0.31	0.759
Psychological distress	24.17 (7.27)	24.42 (7.35)	−0.30	0.766
Child externalizing behaviors T3	67.01 (7.82)	67.52 (7.82)	−0.57	0.567

*Bolded indicates statistically significance, p < 0.05*.

**Table 2:** Associations between child externalizing problems, child temperament, and maternal parenting.

**Variables**	**1**	**2**	**3**	**4**	**5**	**6**	**7**	**8**
1. Externalizing behaviors	–							
2. Negative affectivity	0.363	–						
3. Effortful control	−0.299	−0.083 *ns*	–					
4. Surgency/extraversion	0.305	0.034 *ns*	−0.146	–				
5. Hostility	0.332	0.180	−0.247	0.196	–			
6. Neglect	0.242	0.142	−0.249	0.113	0.568	–		
7. Warmth	−0.187	−0.106 *ns*	0.281	−0.116	−0.399	−0.617	–	
8. Psychological distress	0.311	0.179	−0.095 *ns*	0.145	0.453	0.410	−0.256	–
Mean (SD)	67.22 (7.81)	4.42 (0.79)	4.80 (0.64)	4.95 (0.84)	24.93 (5.67)	19.98 (4.05)	74.62 (5.41)	24.26 (7.30)
Min-max	34–84	2.45–6.26	2.62–6.31	2.16–6.96	15–47	15–37	32–80	14–51

*All statistically significance with p < 0.05 unless indicated otherwise (ns, not significant)*.

**Figure 1:**
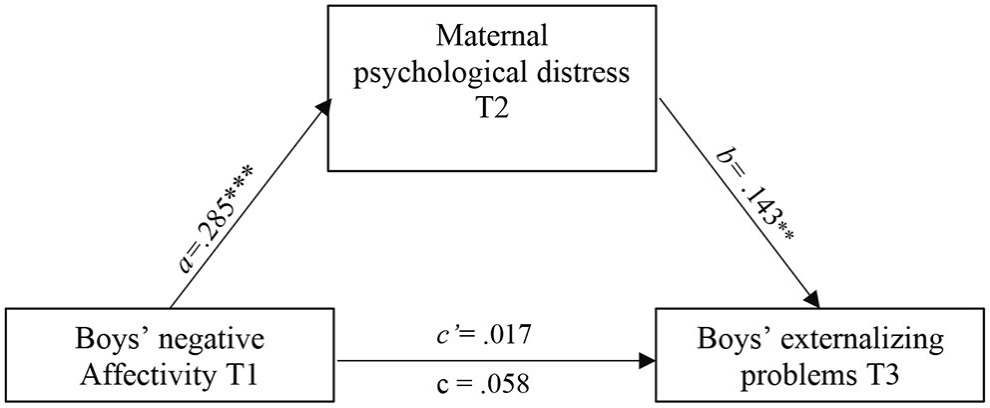
Mediation of psychological distress in the association between boys' negative affectivity with a small a and externalizing problems. All estimates are standardized. ** *p* < 0.01. *** *p* < 0.001.

**Figure 2:**
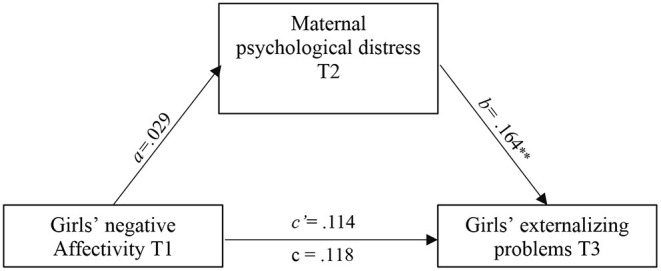
Mediation of psychological distress in the association between girls' negative affectivity with a small a and externalizing problems. All estimates are standardized. ** *p* < 0.01.

According to CFI, TLI and RMSEA indexes, all path analysis models presented a good fit. Table 3 shows the standardized path estimates of the total, direct, and indirect associations between child negative affectivity, maternal parenting and psychological distress, and externalizing problems. Results revealed only one significant indirect effect *via* maternal psychological distress. Specifically, while child negative affectivity was not significantly associated with child externalizing problems, the standardized indirect effect through maternal psychological distress was significant (β = 0.028, SE = 0.011, [CI = 0.009, 0.055]), explaining 38.9% of the total effect. Furthermore, this indirect effect was moderated by the child's sex (CFI = 0.995, TLI = 0.987 and RMSEA = 0.017 [0.000, 0.088]). As shown in Figures 1, 2, the indirect link between negative affectivity and externalizing problems *via* maternal psychological distress was significant for boys (β = 0.285, SE = 0.076, *p* = 0.000), but not for girls (β = 0.029, SE = 0.096, *p* = 0.764). No mediated or indirect link between child negative affectivity and externalizing problems *via* maternal hostility, neglect, or warmth was revealed.

**Table 3:** Mediation of maternal parenting and psychological distress in the association between negative affectivity and externalizing problems.

	**Mediation**						**Model fit**					
	**Total effect with no mediator**	**Direct effect with mediator**	**Indirect effect**	**a-link**	**b-link**	**c^**′**^-link**	**χ^2^**	***df***	***p***	**CFI**	**TLI**	**RMSEA**
Hostility	0.061 (0.045)	0.051 (0.045)	0.010 (0.005)	0.080 (0.049)	0.123 (0.043)*	0.051 (0.045)	10.55	6	0.103	0.978	0.945	0.048 [.000, 0.095]
Neglect	0.059 (0.045)	0.056 (0.045)	0.003 (0.003)	0.046 (0.045)	0.073 (0.039)	0.056 (0.045)	9.19	6	0.163	0.985	0.962	0.041 [.000, 0.089]
Warmth	0.066 (0.045)	0.062 (0.045)	0.004 (0.005)	−0.090 (0.048)	−0.050 (0.048)	0.062 (0.045)	10.46	6	0.106	0.977	0.942	0.048 [.000, 0.095]
Psychological distress	0.072 (0.045)	0.044 (0.045)	0.028 (0.011)*	0.179 (0.060)**	0.155 (0.040)***	0.044 (0.045)	12.69	6	0.048	0.966	0.916	0.059 [.005, 0.104]

All estimates in parentheses correspond to standard errors. All estimates are standardized, therefore, coefficients in this table correspond to effect sizes. ^*^p < 0.05, ^**^p < 0.01, ^***^p < 0.001.

Next, we examined the standardized path estimates of the total, direct, and indirect associations between child surgency/extraversion, maternal parenting and psychological distress, and externalizing problems. As shown in Table 4, child surgency/extraversion directly predicted externalizing problems, with total effects accounting for 13–14% of the variance. Maternal psychological distress significantly mediated this association (β = 0.021, SE = 0.010, [CI = 0.005, 0.045]), explaining 15.3% of the total effect. Similarly, maternal hostility significantly mediated the association between child surgency/extraversion and externalizing problems (β = 0.015, SE = 0.009, [CI = 0.001, 0.045]), explaining 11.3% of the total effect. These mediation models were not moderated by child sex, suggesting a similar pattern of associations for boys and girls. No indirect or mediated link between child surgency/extraversion and externalizing problems *via* maternal neglect or warmth was revealed.

**Table 4:** Mediation of maternal parenting and psychological distress in the association between surgency/extraversion and externalizing problems.

	**Mediation**						**Model fit**					
	**Total effect with no mediator**	**Direct effect with mediator**	**Indirect effect**	**a-link**	**b-link**	**c^**′**^-link**	**χ^2^**	***df***	***p***	**CFI**	**TLI**	**RMSEA**
Hostility	0.133 (0.047)**	0.118 (0.046)**	0.015 (0.009)*	0.133 (0.061)*	0.113 (0.041)*	0.118 (0.046)*	10.64	6	0.100	0.978	0.944	0.049 [.000, 0.096]
Neglect	0.130 (0.047)**	0.125 (0.047)**	0.007 (0.005)	0.085 (0.061)	0.069 (0.036)	0.125 (0.047)**	9.08	6	0.169	0.985	0.963	0.040 [.000, 0.089]
Warmth	0.130 (0.047)**	0.127 (0.046)**	0.003 (0.004)	−0.064 (0.056)	−0.044 (0.049)	0.127 (0.046)**	11.82	6	0.066	0.970	0.924	0.055 [.000, 0.101]
Psychological distress	0.137 (0.047)**	0.116 (0.047)*	0.021 (0.010)*	0.144 (0.062)*	0.149 (0.040)***	0.116 (0.047)*	15.45	6	0.017	0.953	0.883	0.070 [.027, 0.114]

All estimates in parentheses correspond to standard errors. All estimates are standardized, therefore, coefficients in this table correspond to effect sizes. ^*^p < 0.05, ^**^p < 0.01, ^***^p < 0.001.

Lastly, we examined the standardized path estimates of the total, direct, and indirect associations between child effortful control, maternal parenting and psychological distress, and externalizing problems. Table 5 shows that higher child effortful control directly predicted lower levels of externalizing problems, with total effects accounting for 12–13% of the variance. Maternal psychological distress significantly mediated this association (β = −0.017, SE = 0.010, [CI = −0.042, −0.002]), explaining 13% of the total effect. This mediation model was not moderated by the child's sex. No indirect or mediated link between child effortful control and externalizing problems *via* maternal hostility, neglect or warmth was revealed.

**Table 5:** Mediation of maternal parenting and psychological distress in the association between effortful control and externalizing problems.

	**Mediation**						**Model fit**					
	**Total effect with no mediator**	**Direct effect with mediator**	**Indirect effect**	**a-link**	**b-link**	**c^**′**^-link**	**χ^2^**	***df***	***p***	**CFI**	**TLI**	**RMSEA**
Hostility	−0.117 (0.045)**	−0.115 (0.045)**	−0.002 (0.009)	−0.012 (0.069)	0.123 (0.040)**	−0.115 (0.045)**	12.21	6	0.057	0.970	0.924	0.056 [.000, 0.102]
Neglect	−0.117 (0.045)**	−0.119 (0.045)**	0.002 (0.005)	0.029 (0.067)	0.076 (0.035)*	−0.119 (0.045)**	9.89	6	0.129	0.982	0.954	0.045 [.000, 0.093]
Warmth	−0.120 (0.046)**	−0.113 (0.045)**	−0.007 (0.014)	0.295 (0.057)***	−0.025 (0.050)	−0.113 (0.045)**	5.38	6	0.496	1.00	1.00	0.000 [.000, 0.068]
Psychological distress	−0.130 (0.045)**	−0.113 (0.045)**	−0.017 (0.010)*	−0.106 (0.055)*	0.156 (0.039)***	−0.113 (0.045)*	17.90	6	0.007	0.943	0.858	0.078 [.038, 0.122]

All estimates in parentheses correspond to standard errors. All estimates are standardized, therefore, coefficients in this table correspond to effect sizes. ^*^p < 0.05, ^**^p < 0.01, ^***^p < 0.001.

Overall, child temperament factors explained most of the variance of externalizing problems, even if maternal psychological distress (and maternal hostility for the model with child surgency/extraversion) partly explained these associations.

## Discussion

The current study indicates a child temperament-driven effect on maternal psychological distress and hostility, which in turn, predicts externalizing problems among a clinical population of children with conduct problems. By showing the effects of child temperament on levels of externalizing problems directly and indirectly *via* adverse maternal parenting (hostility) and psychological distress, our findings lend support to the transactional perspective.

Specifically, greater maternal psychological distress contributed to higher levels of externalizing problems among boys with higher negative affectivity; an indirect model that was not found for girls. This finding suggests differential susceptibility to externalizing problems among boys with negative affectivity confronted with maternal psychological distress. A potential explanation may hinge on mothers' expectations about how their children should behave based on their gender schema, and their acceptance (or lack thereof) of these behaviors. Considering that negative affectivity is more commonly reported by mothers (and perhaps more socially accepted) of girls than boys (Olino et al., 2013; a finding corrborated in the present study), mothers of boys exhibiting negative affectivity may have difficulty accepting their son's negative emotions, resulting in greater maternal psychological distress.

As for child surgency/extraversion, this temperamental factor was directly associated with the development of externalizing problems. Furthermore, this association was partially mediated by maternal hostility and psychological distress. Children with high levels of surgency/extraversion are likely to be overly outwardly engaged, while simultaneously exhibiting a general disregard for social rules and boundaries. They have been found to use aggressive strategies to overcome barriers or limits when seeking something that is perceived as highly rewarding and to manifest frustration when goals are denied (Berdan et al., 2008). They are also likely to exhibit impulsive, risk taking, and seeking sensation behaviors (Rothbart, 2012). Child surgency/extraversion, contrary to effortful control and negative affectivity, may thus be linked with parental behaviors management. Indeed, mothers of children with high levels of surgency/extraversion may be more likely to resort to hostility or coercion to restrain their child's difficult to manage behaviors. They may also feel powerless or overwhelmed in the face of their child's challenging and risky behaviors, leading to greater psychological distress. On the other hand, effortful control is narrowly linked to cognition and executive functions (Bridgett et al., 2013), and negative affectivity is closely related to emotional self-regulation (Uhl et al., 2019), which may explain why these temperamental factors did not elicit higher maternal hostility.

Maternal psychological distress also explained the direct association between lower child effortful control and greater child externalizing problems. In our study, maternal psychological distress included symptoms of depression, anxiety, irritability, and cognitive problems. In support of our finding, child attention and emotion regulatory difficulties have been previously linked to maternal anxiety (Tsotsi et al., 2021). To extend these findings, future studies could focus on the role of specific maternal psychological distress symptoms to better understand their unique indirect effects in the associations between child temperament factors and externalizing problems.

Interestingly, neglectful parenting and maternal warmth did not significantly explain associations between child temperament and externalizing problems. While indirect models were not identified, our findings do not exclude the possibility that maternal neglect and warmth may interact with child temperament to predict child externalizing problems. For instance, one study revealed that children with high levels of negative affectivity had higher externalizing problems when exposed to low quality parenting (Stoltz et al., 2017). Similarly, a meta-analysis demonstrated that children with negative emotionality during infancy were more vulnerable to externalizing problems when confronted to negative parenting, but also profited more from positive parenting (Slagt et al., 2016). Such findings were not found for surgency/extraversion or effortful control (Slagt et al., 2016).

Taken together, the present study provides answers to important questions regarding temperament-driven effects on externalizing problems among children with early onset conduct problems. Children's temperament explained most of the variance in the prediction of externalizing problems, controlling for several covariates including the child's initial level of externalizing problems. Furthermore, indirect models via maternal psychological distress and hostility were identified, though the strength of these associations was modest. Nevertheless, our results are congruent with the transactional perspective (Sameroff, 2009) in that different child temperamental factors are distinctly associated with adverse maternal characteristics, which are subsequently linked to greater child externalizing problems. As for next steps, research should center on the bidirectional associations between child temperamental factors and maternal parenting practices and distress, as well as interactions between these factors in predicting child externalizing problems, to further disentangle these links. Our finding also revealed one specific mechanism for boys, which supports the differential susceptibility model. Specifically, boys, but not girls, with negative affectivity may be more susceptible to externalizing problems when exposed to maternal psychological distress (Belsky, 2013; Slagt et al., 2016).

Despite these new insights, results should be interpreted with caution. First, given our sample, our results cannot be generalized to children from the general population. Conducting this study on children with conduct problems might also have limited between-person variations in externalizing problems. Future studies on children from the general population could shed light on whether these mechanisms also exist among non-clinically referred children. Second, our measures of temperament, parenting, and externalizing problems were based on maternal reports only, which can introduce shared measurement bias. The longitudinal design of our study, however, lessens this limitation. The use of well-validated and recognized measures in the field of child development (e.g., CBQ-SF, CBCL) further adds to the robustness of study findings. Third, temperamental characteristics were reported during a specific timeframe (i.e., during the past 6 months) and within various contexts which minimized subjectivity. Lastly, the given that child externalizing and internalizing problems can co-occur (McElroy et al., 2018), future research investigating internalizing problems as a confounding variable is warranted.

## Conclusion

The results of the present study support the relevance of temperament-based interventions for children with conduct problems and of increased mental health support for their mothers. By aiding mothers in the development of a larger repertoire of parenting strategies for dealing with children with various temperamental characteristics, mothers may be better equipped to respond appropriately to their child, hence, limiting the development of child externalizing problems.

Our findings also support the need to consider child temperament and maternal parenting and mental health in prevention programs targeting child externalizing problems (Smedler et al., 2015). Programs supported by scientific evidence in preventing child externalizing problems focus on parent training (e.g., Incredible Years and Triple-P), family support (e.g., Family Check-Up), management of classroom behaviors (e.g., Good Behavior Game) or cognitive-based intervention [e.g., Coping Power (Smedler et al., 2015)]. While some of these programs target parenting skills, very few (if none) consider child temperamental characteristics. Such considerations could potentially sustain the long-term effects of these programs (Smedler et al., 2015). In addition, reinforcing the child's ability to adequately express emotion, focus their attention (i.e., reactivity) and regulate their emotions before school entry appear to be effective strategies for preventing externalizing problems in middle-school.

## Data Availability Statement

The data analyzed in this study is subject to the following licenses/restrictions: This dataset is not available outside the secure server where the data is hosted. Requests to access this dataset should be directed to michele.dery@usherbrooke.ca.

## Ethics Statement

This study was reviewed and approved by by Le Comité d'éthique de la recherche—éducation et sciences sociales de l'Université de Sherbrooke (No. 2015-1076, 2015-26-ESS/Dery). Written informed consent to participate in this study was provided by the participants' legal guardian/next of kin.

## Author Contributions

GG-C conceived the project, conducted and oversaw all aspects of the analyses, and wrote the paper. KP conceived the project contributed to the interpretation, wrote-up, and reviewed the manuscript. WG contributed to the analyses, interpretation of the findings, and reviewed the manuscript. J-PL contributed to data collection, the interpretation of the results, and reviewed the manuscript. MD managed the data collection, contributed to data collection, the interpretation of the results, and reviewed the manuscript. All authors contributed to the article and approved the submitted version.

## Funding

Data collection of this longitudinal study was supported by grants from the Canadian Institutes of Health Research (Grant NRF 82694) and the Social Sciences and Humanities Research Council (Grant SSHRC 37890). GG-C research is supported by the Canada Research Chair programs.

## Conflict of Interest

The authors declare that the research was conducted in the absence of any commercial or financial relationships that could be construed as a potential conflict of interest.

## Publisher's Note

All claims expressed in this article are solely those of the authors and do not necessarily represent those of their affiliated organizations, or those of the publisher, the editors and the reviewers. Any product that may be evaluated in this article, or claim that may be made by its manufacturer, is not guaranteed or endorsed by the publisher.
